# Brain Responses to Words in 2-Year-Olds with Autism Predict Developmental Outcomes at Age 6

**DOI:** 10.1371/journal.pone.0064967

**Published:** 2013-05-29

**Authors:** Patricia K. Kuhl, Sharon Coffey-Corina, Denise Padden, Jeffrey Munson, Annette Estes, Geraldine Dawson

**Affiliations:** 1 Institute for Learning & Brain Sciences, University of Washington, Seattle, Washington, United States of America; 2 Center for Mind and Brain, University of California Davis, Davis, California, United States of America; 3 Department of Psychiatry, University of Washington, Seattle, Washington, United States of America; 4 Department of Speech and Hearing Sciences, University of Washington, Seattle, Washington, United States of America; 5 Department of Psychiatry, University of North Carolina, Chapel Hill, North Carolina, United States of America; 6 The Autism Speaks Foundation, New York, New York, United States of America; University of Jyväskylä, Finland

## Abstract

Autism Spectrum Disorder (ASD) is a developmental disability that affects social behavior and language acquisition. ASD exhibits great variability in outcomes, with some individuals remaining nonverbal and others exhibiting average or above average function. Cognitive ability contributes to heterogeneity in autism and serves as a modest predictor of later function. We show that a brain measure (event-related potentials, ERPs) of word processing in children with ASD, assessed at the age of 2 years (N = 24), is a broad and robust predictor of receptive language, cognitive ability, and adaptive behavior at ages 4 and 6 years, regardless of the form of intensive clinical treatment during the intervening years. The predictive strength of this brain measure increases over time, and exceeds the predictive strength of a measure of cognitive ability, used here for comparison. These findings have theoretical implications and may eventually lead to neural measures that allow early prediction of developmental outcomes as well as more individually tailored clinical interventions, with the potential for greater effectiveness in treating children with ASD.

## Introduction

Autism Spectrum Disorder (ASD) is a severe and pervasive disorder of neurodevelopment that emerges early and typically results in lifelong disability [1]. ASD is a heterogeneous syndrome—individuals vary widely in the degree of impairment in the core areas of language and social function. Substantial variability also exists in cognitive and adaptive function. Increasingly, research is focused on the identification of reliable early measures that predict future function in children with ASD. We report here that in a prospective, longitudinal study of children with ASD, a brain measure of early word processing at the age of 2 years predicts individual variation in linguistic, cognitive, and adaptive behavior 2 and 4 years later, at 4 and 6 years of age. Research that identifies robust predictors of an individual child's developmental outcome has theoretical implications as well as the potential for improving clinical prognosis and for developing novel interventions tailored to specific learning needs.

Mixed and sometimes contradictory results are reported in regard to early prediction of later developmental outcomes in children with ASD. Although group-level improvements are observed over time, outcome for individual children varies widely. Charman and colleagues [2] reported increasing variance in behavioral measures in children with ASD over time, due to improvement in some individuals and stable or declining performance in others. Previous longitudinal studies of young children with ASD indicate that cognitive ability is one of the most salient factors contributing to heterogeneity in autism [3]. Predictive relationships between verbal and nonverbal cognitive ability in childhood and later function in ASD have been widely reported [4]–[9]. There is strong interest in identifying reliable brain-based predictors of outcome in ASD that (a) improve upon these existing predictors, (b) can be assessed very early in development, and (c) offer the potential of contributing to theory and clinical practice.

Previous work in our laboratory, both in children with ASD and in typically developing (TD) children, indicates that brain measures of early language processing are promising candidates as potential predictors of outcome in children with ASD. In a previous study, we demonstrated that event-related potentials (ERPs) in response to speech provided a sensitive index of speech processing in children with ASD [10]. The study investigated a component of the ERP, the mismatch negativity (MMN), which indicates syllable discrimination (see [11] for review) in preschool-aged children with ASD and TD controls. The results revealed the expected MMN for TD controls; however, the MMN was not observed at the group level in children with ASD. Subsequently, we dichotomously classified the children with ASD based on a well-studied social variable, the presence or absence of an auditory preference for ‘motherese’ (e.g., [12], [13] for review) as opposed to tonal analogs of the motherese signals. When the ERP results of the children with ASD were re-examined based on the sub-grouping, the ASD subgroup that preferred motherese produced ERP responses to speech syllables that were similar to the TD control group, whereas the ASD subgroup that preferred the tonal analog of the motherese signals did not produce an MMN response to the syllable change [10].

In other words, the study showed that using a social measure to classify children with ASD into two groups, one with fairly typical social responses and the other with less typical social responses, resulted in two very distinct patterns of neural response to linguistic stimuli (speech syllables), one which resembled TD children, and the other highly atypical [10]. These results served as the impetus to further extend our theoretical work linking linguistic processing to social factors, which has been conducted in this laboratory over the last decade in TD children [14]–[17], to children with ASD. In addition, the findings of Kuhl et al. [10] provided the motivation and hypotheses guiding the current study.

The current study involved two Phases. Phase 1 extends the ERP results of Kuhl et al. [10] in children with ASD from phonetic stimuli in preschool-aged children to word processing in younger children with ASD—examining whether the same subgroup effects are observed (i.e., affected children with less severe social symptoms exhibit an ERP response to words that is similar to TD children, while the subgroup with more severe social symptoms exhibit an atypical neural response). Phase 2 builds upon the results of Phase 1, investigating the consequences of the neural response to words at age 2 years in a prospective longitudinal study. In Phase 2, we evaluate the predictive power of the more typical ERP response to words in children with ASD (identified in Phase 1) in terms of future linguistic, cognitive, and adaptive function in the *full* group of children with ASD. In brief, Phase 1 studies the relationship between word processing and concurrent social function. Phase 2 builds on the results of Phase 1, evaluating the predictive power of a more typical neural response to words on later linguistic, cognitive, and adaptive function.

To achieve the goal of Phase 1, we used an ERP word paradigm that is well documented in TD children. The ERP word paradigm examines responses to known, unknown, and backward words and has produced stable patterns of response from TD children across laboratories [18]–[23]. Collectively, these studies show that the pattern of ERP response to known and unknown words follows a specific developmental time course related to age and language proficiency. Mills, Coffey-Corina and Neville [18], [19] separated TD children between 13 and 20 months of age into subgroups based on parental reports of word comprehension and word production. They demonstrated that the overall characteristics of the ERP response to known and unknown words differ with age and with language proficiency. In the youngest and least proficient children, significant differences in ERP responses to known and unknown words are broadly and bilaterally distributed. With increasing age, and with increasing language ability at a given age, significant differences in ERP responses to known and unknown words are limited to the temporal and parietal regions of the left hemisphere [18], [19]. Mills and colleagues [18], [19] also evaluated ERPs to a complex auditory signal (i.e., known words played backward) and found a broad positive response that did not differ with age or language ability. They interpreted the developmental pattern of results as evidence that the functional organization of language-relevant brain systems becomes progressively more specialized with increasing language abilities, and is specific to differences in word meaning—not simply a differential response to words and complex auditory stimuli.

The pattern of developmental change over time shown by Mills and her colleagues has been replicated [20] and extended in investigations of known words and phonetically similar nonsense words [20], known words and novel words just learned by toddlers [21], known and unknown words in bilingual populations [22], and known and unknown words in late talkers up to age 30 months [23]. All studies report the same pattern of ERP response to known and unknown words as a function of age and language proficiency, supporting the interpretation that language proficiency, and not age or brain structure maturation, accounts for the signature changes in language-relevant brain activity from broadly and bilaterally distributed to focal brain activity limited to the left temporal and parietal regions.

In the current study, Phase 1 tested the hypothesis that the ERP pattern of results in response to words in TD children and in two groups of children with ASD classified dichotomously on a social variable would reveal the same group and subgroup effects we had previously seen with speech syllables [10]. In other words, we expected that children with ASD who had less severe social symptoms would produce ERP responses to words resembling the TD controls, while affected children with more severe social symptoms would produce a very different pattern.

Phase 2 depended upon the results of Phase 1. We reasoned that if the results of Phase 1 produced the expected group and subgroup effects—showing that affected children with less severe social symptoms produce an ERP response to words resembling the TD controls—Phase 2 would examine the predictive power of the defining characteristic of that more typical ERP response to words in terms of future language, cognitive, and adaptive function in the *full* group of children with ASD. Phase 2 was motivated by studies conducted across laboratories in TD children which show future language is predicted by both ERP brain measures of early speech processing [24]–[27] and behavioral measures of early speech processing [28]–[33]. For example, the response to speech syllables at 7 months of age predicts the speed of language growth to 30 months of age [24], and also predicts measures of reading readiness at the age of 5 years [28], [29]. Given that research in TD children shows that neural measures of speech processing provide valid indicators of language growth, we reasoned that the use of neural measures to characterize language processing in children with ASD would provide a highly sensitive index of the brain functions underlying language learning, which would in turn provide an excellent predictor of functional outcomes in these children.

Phase 1 of the current study investigated children with ASD at enrollment in an intervention study at 2 years of age (Time 1) [34], employing an ERP measure of word processing with a documented developmental pattern in TD children, as well as a concurrent measure of severity of social symptoms. Following Kuhl et al. [10], we categorized children with ASD on the social variable, and hypothesized that the association between the neural response to speech and classification based on social function [10] would again be observed: Brain responses in children with ASD exhibiting less severe social symptoms would be similar to TD controls (i.e., significant differences between known and unknown words limited to left temporal/parietal electrode sites), whereas brain responses in children with ASD who have more severe social symptoms would be atypical. Phase 2 of the current study investigated the potential for the defining characteristic of this neural response to words in 2-year-old children with ASD to, in turn, serve as an early brain indicator of functional outcome in the areas of language, cognition, and adaptive behavior 2 years later at the end of the experimental intervention when children were 4 years old (Time 2) [34], and 4 years later when children were 6 years old (Time 3). We hypothesized that the defining characteristic of the more typical ERP response to words at the temporal/parietal electrode sites would have implications for future functional outcomes in both subgroups of children with ASD, independent of cognitive function at Time 1.

## Methods

### Ethics Statement

The paper reports data from human subjects, and ethical approval was obtained from the University of Washington Human Subjects Division. Written informed consent was obtained from parents or legal guardians of all participants according to the principles explained in the Declaration of Helsinki, and the rights of these participants were protected.

### Participants

Participants were recruited from local parent advocacy groups, Washington State Department of Developmental Disabilities, clinics, hospitals and the University of Washington Communications Studies Participant Pool.

#### Children with ASD

Children with ASD in the present study were drawn from a larger group of children enrolled in a previously reported randomized, controlled trial that demonstrated the efficacy of the Early Start Denver Model (ESDM) as a developmental behavioral intervention (ClinicalTrials.gov, NCT00090415) [34]. The inclusion criteria were: age below 30 months at entry, meeting criteria for autistic disorder on the Toddler Autism Diagnostic Interview [35], meeting criteria for autism or ASD on the Autism Diagnostic Observation Schedule [36], a clinical diagnosis based on *Diagnostic and Statistical Manual of Mental Disorders, Fourth Edition* (DSM-IV) criteria [37] using all available information, residing within 30 minutes of the University of Washington, and willingness to participate in a 2-year intervention. See Dawson et al. [34] for detailed information. Data were collected from children with ASD at enrollment in the clinical trial (Time 1), at the end of the 2-year intervention phase (Time 2), and in a follow up study conducted 2 years after the completion of the intervention portion of the clinical trial (Time 3).

Useable ERP data were collected from 24 of the children with ASD at Time 1. These children were between 1.6 and 2.6 years old (M = 2.1 years, SD = .3), 17 males and 7 females. Usable data were not obtained from the remaining children with ASD due to: less than 20 artifact-free ERP trials for each word type (N = 8), refusal to wear the Electro-cap (N = 8), English not the primary language spoken in the home (N = 6), declined ERP testing (N = 3), and seizure activity following enrollment (N = 1). Affected children with usable ERP data did not differ from affected children without usable ERP data in terms of age, gender, diagnostic measures or standardized measures of receptive language cognitive ability, or adaptive behavior at Time 1.

Children with ASD were divided into subgroups based on a median split of their social scores on the Autism Diagnostic Observation Schedule (ADOS) [36] to investigate the relationship between ERPs to words and severity of social symptoms: those exhibiting more severe social symptoms and those exhibiting less severe social symptoms. All children were evaluated with ADOS-WPS, Module 1: social total median = 11.5, range = 6–14 (Table 1). Subgroups did not differ significantly in chronological age. Although children exhibiting more severe social symptoms had lower mean scores on standardized behavioral measures of language, cognitive ability, and adaptive function, these differences were marginally significant for language measures, and were not significant for cognitive ability and adaptive behavior (Table 1).

**Table 1:** Time 1 diagnostic and standardized behavioral measures for subgroups of children with ASD.

	Less Severe Social Symptoms N = 12			More Severe Social Symptoms N = 12					
	Mean	SD	Range	Mean	SD	Range	F	MS	P
**ADOS Social Total**	8.9	1.4	6–11	12.9	0.9	12–14	70.79	96.00	0.000
**Age (years)**	2.1	0.3	1.7–2.6	2.1	0.3	1.6–2.5	0.00	0.00	0.947
**PLS Aud Comprehension**	68.8	14.8	50–102	58.8	8.5	50–77	4.20	610.04	0.053
**PLS Exp Communication**	76.5	12.5	50–92	65.8	15.8	50–106	3.38	682.67	0.080
**Mullen Composite**	67.3	8.8	53–85	60.0	13.0	49–95	2.56	315.38	0.124
**Vineland Composite**	70.6	4.9	62–78	68.8	7.5	57–85	0.46	18.38	0.503

Families of 22 of the 24 children with ASD agreed to participate in the clinical trial following randomized assignment to intensive intervention groups. These 22 children with ASD returned for diagnostic testing when they were at least 33 months old, and all retained the diagnostic status as described above. Two participants withdrew during the 2-year intervention period (1 female) before Time 2 data collection, and one male participant did not return for the follow up study at Time 3.

#### Typically developing controls

Twenty TD children were enrolled as control subjects for this study. These children were between 1.6 and 2.6 years old (M = 2.1 years, SD = .3), 13 males and 7 females. Families reported no speech, language, hearing or other developmental problems in the participants or in their siblings. An additional 10 TD children were excluded due to: insufficient numbers of ERP trials (N = 3), refusal to wear the Electro-cap (N = 5), and equipment problems during ERP recordings (N = 2). Diagnostic and standardized behavioral measures were not collected from the TD control group.

### ERP Methods

#### ERP Word Stimuli

The stimuli were 10 different words of each type: words the child comprehended (‘known words’), words the child did not comprehend (‘unknown words’), and the comprehended words presented backward (‘backward words’). Known words were early acquired nouns selected from the MacArthur Communicative Development Inventory norming study [38]. Unknown words were low frequency words matched for number of syllables and were similar in phonological form to known words. As in previous ERP word studies in young children [18], [19], [22], [23], we prepared a large pool of candidate known words naturally spoken by a female voice; digitized at 16 bits, 44 kHz sampling rate; and loudness balanced.

Individualized lists were determined for each child during the week before ERP testing based on parental report. Parents completed a questionnaire, indicating whether each potential known word was (1) produced, or (2) comprehended. For children with ASD, we began with a standard list and then substituted additional words to insure that each word was comprehended by the child, and to maximize the inclusion of produced words on the individualized list. We also maximized the overlap between the word lists of ASD and TD children, creating word lists for TD controls to match the lists of children with ASD who had already completed ERP data collection. However, children were recruited and scheduled to meet the needs of the randomized controlled trial which prevented one to one matching. ERP word lists for the present study included 50 known words (mean duration = 671 ms) and 34 unknown words (unknown word mean duration = 745 ms). Frequency of presentation of specific known and unknown words was similar among the 3 groups (affected children with more severe social symptoms, affected children with less severe social symptoms, and TD controls), with intergroup word frequency correlations ranging from .815 to .903 (*p*<.0005).

#### ERP Procedure

EEG was collected continuously from 20 electrode sites using the standard International 10–20 system. Participants wore an elastic Electro-cap, and data were acquired using SAI amplifiers, band pass filtered at .1–60 Hz. Electrode impedances were kept below 12 kΩ. The left mastoid served as reference. Participants listened passively while sitting on their parent's lap, watching an assistant playing quietly with toys while a silent child-oriented video was displayed on a monitor behind the assistant. Stimuli were presented from two speakers placed on either side of the video monitor, approximately 4 feet in front of the participant. Words were presented at 65–67 dBA. The inter-stimulus interval was 2000 ms, onset to onset. All data were processed off-line, using epochs of 100 ms pre-stimulus and 1000 ms post-stimulus onset. Trials exceeding the threshold of positive or negative 100 microvolts were rejected. In addition, hand editing was performed to ensure data were free of eye movements, muscle artifact and drift. Finally, individual subject averages were filtered using a low pass filter with a cut off of 25 Hz. The average number of trials per word-type was 42.7 (SD = 8.1) for TD controls, 40.6 (SD = 5.2) for affected children with more severe social symptoms, and 50.1 (SD = 12.5) for affected children with less severe social symptoms. Individual and grand mean ERPs were examined to determine the most appropriate interval consistent with that found to be sensitive to differences in word type by Mills and colleagues [18]–[23]. The mean amplitude of waveforms was measured between 200–500 ms at lateral electrode sites (F3/4, F7/8, T3/4, C3/4, and P3/4) chosen to approximate the customized electrode array employed in the previous ERP studies using this paradigm [18]–[23]. This previous work employed the standard International 10–20 electrode array at frontal (F7/8) and occipital (O1/2) electrode sites, and a custom electrode array at anterior temporal (50% of the distance from F7/8 and T3/4), temporal (33% of the distance from T3/4 to C3/4) and parietal (50% of the distance between T3/4 and P3/4) electrode sites.

### Standardized Behavioral Measures

Standardized measures of receptive language, cognitive ability, and adaptive behavior were administered to children with ASD at three time points: at enrollment in the intervention study (Time 1), 2 years later at the end of the experimental intervention (Time 2), and 4 years later in a follow up study (Time 3). TD controls did not complete standardized behavioral measures. Children were evaluated by experienced examiners naïve to intervention status and all tests yielded norm-referenced standard scores. The standardized measures of receptive language and cognitive ability used in the intervention study had age range limitations requiring different standardized measures for these domains in the follow up study at Time 3. In addition, not all participants at Time 3 completed all behavioral measures: 18 participants completed the standardized measure of receptive language, 19 participants completed the standardized measure of cognitive ability, and 15 participants completed the standardized measure of adaptive behavior. Specific standardized tests at each time point are listed in Table 2.

**Table 2:** Standardized measures of receptive language, cognitive ability, and adaptive behavior.

	*Time 1 (2 years) & Time 2 (4 years)*	*Time 3 (6 years)*
**Receptive Language**	PLS4 Aud. Comprehension Std. Score [39]	PPVT4 Standard Score [42]
**Cognitive Ability**	Mullen Composite Standard Score [40]	DAS2 Composite Standard Score [43]
**Adaptive Behavior**	Vineland Composite Standard Score [41]	Vineland Composite Standard Score [41]

Intercorrelations among standardized behavioral measures of receptive language, cognitive ability, and adaptive behavior were significant at each time point; significant among behavioral measures at Time 1 and Time 2 using the same standardized tests for cognitive ability and adaptive behavior; and significant among all behavioral measures at Time 2 and Time 3, using different standardized tests of cognitive ability and receptive language. However, intercorrelations among behavioral measures at Time 1 and Time 3 are not significant, indicating a decline in association over time that is unrelated to the specific standardized tests (Table 3).

**Table 3:** Intercorrelations among outcome measures.

	T1 PLS4	T1 Mullen	T1 Vineland	T2 PLS4	T2 Mullen	T2 Vineland	T3 PPVT	T3 DAS2
**T1 Mullen**	.579(**)							
**T1 Vineland**	.521(**)	819(***)						
**T2 PLS4**	.333	.585(**)	479(*)					
**T2 Mullen**	.159	.579(**)	.635(**)	821(***)				
**T2 Vineland**	.132	.695(**)	.629(**)	715(***)	.790(***)			
**T3 PPVT**	.262	.463	.410	.818(***)	.668(**)	.695(**)		
**T3 DAS2**	.101	.422	.418	.808(***)	.808(***)	.683(**)	.845(***)	
**T3 Vineland**	.080	.390	.293	.750(**)	.790(***)	.774(**)	.766(**)	.871(***)

* p<.05,

** p<.01,

*** p<.001.

As anticipated and consistent with previous research, group-level improvements in standardized behavioral test scores were found in children with ASD over time (Table 4), with significant increases from Time 1 to Time 3 for measures of receptive language, *F*(1, 17) = 32.240, *p* = .000, η_p_^2^ = .655, cognitive ability, *F*(1, 18) = 25.583, *p* = .000, η_p_^2^ = .614, and adaptive function, *F*(1, 14) = 6.021, *p* = .028, η_p_^2^ = .301. Variability also increased from Time 1 to Time 3 as evidenced by increased standard deviations and range of scores (Table 4).

**Table 4:** Age and standardized behavioral measures at Time 1, Time 2, and Time 3.

	Time 1, N = 24			Time 2, N = 20			Time 3*		
	Mean	SD	Range	Mean	SD	Range	Mean	SD	Range
**Age (years)**	2.1	0.3	1.6–2.6	4.3	0.3	4.0–4.8	6.1	0.2	6.0–6.8
**Receptive Language**	63.8	12.9	50–102	83.0	23.0	50–115	94.5	22.9	37–123
**Cognitive Ability**	63.6	11.5	49–95	74.5	21.8	49–125	87.9	23.6	37–118
**Adaptive Function**	69.7	6.2	57–85	65.0	14.9	44–109	78.7	18.0	47–119

* N = 18 for Receptive Language, N = 19 for Cognitive Ability, N = 15 for Adaptive Function.

### Intervention Groups

Families of 22 of the 24 children with useable ERP data agreed to participate in the clinical trial and were randomly assigned to one of two intensive treatment groups as part of the clinical trial. During the two year study enrollment period, the Early Start Denver Model (ESDM) experimental intervention group received an average of 15.2 hours/week (SD = 1.4) of therapist-delivered intervention. In addition, parents reported an additional 16.3 hours/week (SD = 6.2) of interaction using ESDM strategies and 5.2 hours/week (SD = 2.1) in other therapies (e.g., speech therapy, developmental preschool). Families assigned to the Community Intervention (CI) treatment group were referred to local providers for interventions commonly available in the community, and reported an average of 9.1 hours/week of individual therapy and an average of 9.3 hours/week of group interventions (e.g., developmental preschool) [34].

Although the random assignment did not consider availability of usable ERP data or the subgroup based on the severity of social symptoms, the group of children with ASD who had usable ERP data were evenly divided into the two treatment groups: 11 (3 female, 6 with more severe social symptoms) were assigned to the ESDM experimental intervention group, 11 (3 female, 5 with more severe social symptoms) were assigned to the CI treatment group, and 2 (1 female, 1 with more severe social symptoms) declined to participate in the randomized intervention (see [34] for detailed information). Children with useable ERP data assigned to ESDM did not differ significantly from those assigned to the CI group in terms of age, gender, diagnostic measures, standardized behavioral measures, or ERP mean amplitude at Time 1. Nor did they differ significantly on these measures from children without usable ERP data assigned to their respective treatment groups. In addition, toddlers with useable Time 1 ERP exhibited the same significant treatment effects on primary outcome measures (i.e., Time 2 Mullen Composite standard score and Time 2 Vineland Composite standard score) reported by Dawson et al. [34] for the full group of subjects enrolled in the clinical trial.

## Phase 1: ERPs to Words and Social Function

Phase 1 examined whether the ERP response to words in 2-year-old children with ASD who were classified on a social measure and compared to TD controls produced group and subgroup effects similar to those previously observed in a study using speech syllables [10]. We hypothesized that the pattern of ERP brain responses to known and unknown words in children with ASD exhibiting less severe impairments in social function would resemble those of TD controls. That is, we expected to see significant differences between known and unknown words limited to left temporal/parietal electrode sites in these groups, but atypical responses in children with ASD exhibiting more severe impairments. Note that ASD is characterized by deficits in social and linguistic function, and we hypothesized that affected children with ASD would be similar to TD children in the current study, but not that they would be indistinguishable from TD controls.

### Results

Data were analyzed in the same manner as previous ERP word studies of TD children [18]–[23] employing repeated measures ANOVA and planned comparisons at all individual electrode sites measured. ERPs were averaged separately for known, unknown, and backward words, and measurements (200–500 ms time window) were analyzed in ANOVAs with repeated measures of word-type (known, unknown, backward), hemisphere (right, left), and electrode site (F7/8, F3/4, T3/4, C3/4, P3/4). The between subjects factor divided children with ASD based on severity of social symptoms using a median split, just as previous studies divided TD children based on various measures of language proficiency using a median split or percentile rankings [18]–[23]. This approach yielded a three level between subjects group factor: TD, ASD with less severe social symptoms, and ASD with more severe social symptoms. Greenhouse- Geisser corrections were applied when appropriate and partial eta-squared was calculated for main effects and interactions. Planned comparisons were reported as significant at the .05 level.

As anticipated based on previous research [18], [19], mean amplitude of the ERP response to backward words (M = 2.783, SD = 2.052) was significantly more positive than the response to known (M = −1.121, SD = 1.888) and unknown (M = .078, SD = 2.123) words for all groups resulting in a significant main effect for word-type, *F*(2,82) = 11.2, *p* = .000, η_p_^2^ = .215, and no significant main effects for group or significant group by word-type interactions. Since all participants processed backward words in a similar way, consistent with previous research, further analyses were conducted with a two level within subjects word-type factor (i.e., known and unknown words).

Repeated measures analysis of variance compared group (ASD with less severe social symptoms, ASD with more severe social symptoms, and TD) and the repeated factors of word-type (known and unknown), hemisphere, and electrode site, yielding a significant word-type by hemisphere by group interaction, *F*(2, 41) = 5.892, *p* = .006, η_p_^2^ = .223. This three-way interaction reflects group differences in the response to known words. The hemisphere by group interaction is significant for known words, *F*(2, 41) = 7.405, *p* = .002, η_p_^2^ = .265 and is not significant for unknown words, *F*(2, 41) = .147, *p* = .864, η_p_^2^ = .007. Furthermore, the hemisphere by group interaction for known words is driven by children with ASD who have more severe social symptoms—the interaction is significant when comparing affected children with more severe social symptoms to TD controls, *F*(1, 30) = 10.548, *p* = .003, η_p_^2^ = .260, and when comparing affected children with more severe social symptoms to affected children with less severe social symptoms, *F*(1, 22) = 13.696, *p* = .001, η_p_^2^ = .384. The hemisphere by group interaction is not significant when comparing affected children with less severe social symptoms to TD controls, *F*(1, 30) = .699, *p* = .410, η_p_^2^ = .023.

**Figure 1:**
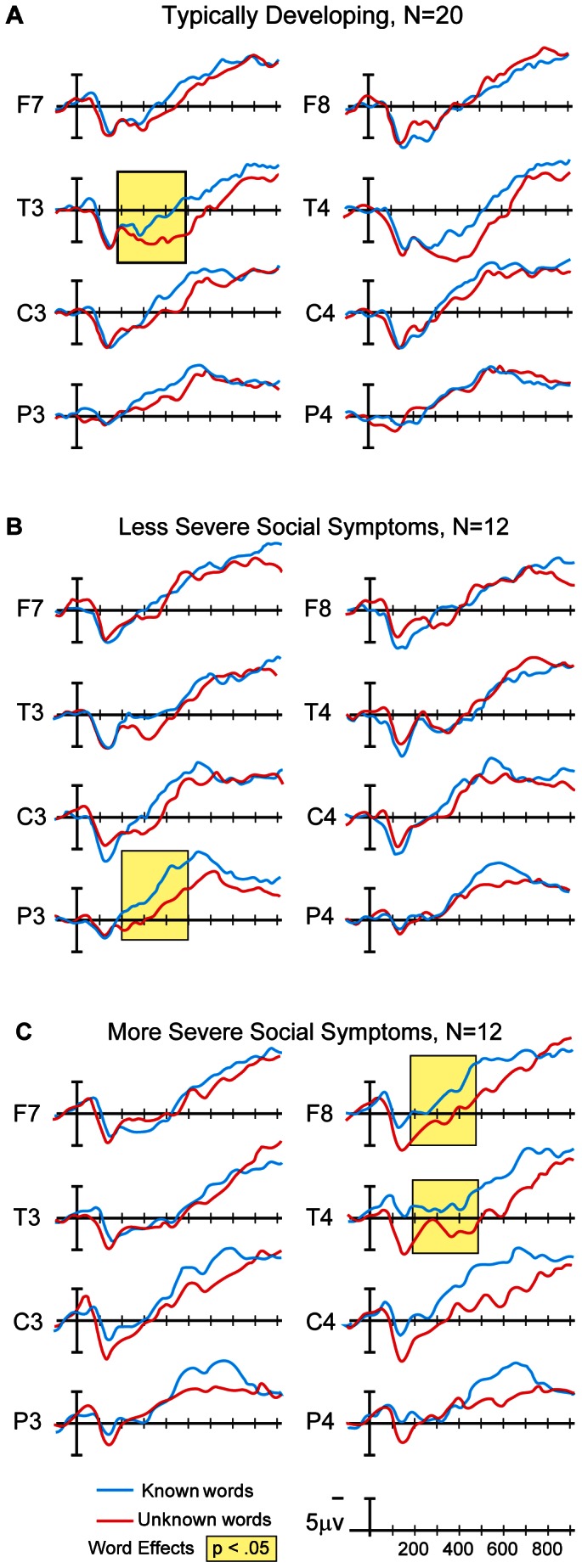
Time 1 ERP waveforms. (A) TD 2-year-olds exhibit a focal response with significant differences between known and unknown words only at the left temporal electrode site T3, (B) children with ASD exhibiting less severe social symptoms show a more typical ERP pattern with a focal response that is significant only at the left parietal electrode site P3, (C) affected children exhibiting more severe social symptoms show a more diffuse response in the right hemisphere.

The ERP word paradigm used in the current study is well documented in TD children [18]–[23]. Previous work conducted planned comparisons at multiple electrode sites and reported significant differences in ERP response to known and unknown words in the temporal and parietal regions of the left hemisphere with increasing age and with increasing language ability at a given age [18], [19]. These studies used a custom electrode array, which defined the temporal electrode site as 33% of the distance from T3/4 to C3/4, and the parietal electrode site as 50% of the distance between T3/4 and P3/4. The current study employed the standard International 10–20 electrode array without custom arrangements of electrode sites. Planned comparisons at each electrode site in the current study revealed that TD children exhibited results consistent with the previously reported signature focal negativity for known words in the left hemisphere. The significant difference in amplitude between known and unknown words was localized to a single left hemisphere electrode site, T3, *F*(1, 19) = 8.302, *p* = .010, η_p_^2^ = .304 (Figure 1A). Children with ASD exhibiting less severe social symptoms also show a focal response at a single left hemisphere electrode site, consistent with previous research and similar to TD controls in the current study, but at the left parietal electrode site, P3, *F*(1, 11) = 7.912, *p* = .017, η_p_^2^ = .418 (Figure 1B). In contrast, ERP waveforms of affected children exhibiting more severe social symptoms show a more diffuse right hemisphere response to words at two electrode sites: T4, *F*(1, 11) = 15.386, *p* = .002, η_p_^2^ = .583; and F8, *F*(1, 11) = 6.680, *p* = .025, η_p_^2^ = .378 (Figure 1C). Taken together, these results indicate a link between the overall pattern of brain response to known words and social function in very young children with ASD: children with less severe social symptoms resemble TD controls, while those with more severe social symptoms show a clearly atypical brain response.

Our results demonstrate that the pattern of response in affected children exhibiting less severe social symptoms resembles that of TD participants in the current study and TD children reported in the literature [18]–[23], with a focal, left hemisphere response in the temporal/parietal region; specifically, significant differences between known and unknown words only at the P3 electrode site. In contrast, affected children exhibiting more severe social symptoms show a more diffuse response with significant word-type effects at 2 right hemisphere electrode sites. Since the median split analysis yielded a relationship between severity of social symptoms and the overall pattern of ERP response across two word-types and 10 electrode sites, we did not anticipate significant associations between ERP mean amplitudes to known or unknown words at individual electrode site and the ADOS social total. And as expected, correlations between the ADOS social total and ERP mean amplitudes to known and unknown words at individual electrode sites were not significant, ranging from −.289 to .196 (p>.10).

However, there is a significant association between the signature focal negativity for known words at the P3 electrode site, when compared to the overall negativity across all electrode sites, and severity of social symptoms. We calculated the average difference between known and unknown words across all electrode sites, and then subtracted that average from the difference between known and unknown words at P3. Children with ASD who exhibit a response to known words (compared to unknown words) that is more negative at the P3 electrode site than the average across all electrode sites have lower ADOS social totals (*r* = .437, *p* = .033). In addition, the ADOS social total is significantly correlated with the concurrent measure of receptive language at Time 1 (*r* = −.543, *p* = .007) and the measure of receptive language obtained 2 years later at Time 2 (*r* = −.509, *p* = .022). The correlation was not significant with the Time 3 measure of receptive language (*r* = −.319, *p* = .197) at age 6. ADOS social total was not associated with measures of cognitive ability or adaptive function at any time point, correlations ranged from −.291 to −.120 (*p*>.10).

Our results thus show significant relationships between a measure of severity of social symptoms in children with ASD (ADOS social total) and our ERP measure of word processing, consistent with our hypothesis of an association between linguistic and social function.

### Discussion

In Phase 1 of the current study, word stimuli were used to test 2-year-old children with ASD who were categorized into two groups based on social function, and comparisons were made with TD controls. TD children show a focal response to known words at a single electrode site in the left temporal/parietal region, consistent with earlier studies employing this ERP word paradigm [18]–[23]. As predicted, children with ASD who exhibit less severe social symptoms show a pattern of ERP response that resembles TD controls – a focal response in the left hemisphere, with one difference. The single electrode site in the temporal/parietal region is T3 for TD controls and P3 for children with ASD, electrode sites consistent with the findings of previous studies. This could potentially indicate a difference in the source of brain activation in response to known words in the two groups. However, ERP methods are not sufficiently sensitive to fully explore this possibility, and we are currently using more sophisticated neuroimaging methods (magnetoencephalography, or MEG) in our laboratory to compare children with ASD and TD controls, which will allow the sources of brain activity to be more accurately localized in both groups.

Children with ASD exhibiting more severe social symptoms show an atypical ERP response to word stimuli, one that is more diffuse and in the right hemisphere, consistent with frequent reports of right hemisphere dominance in ASD, which in turn has been associated with both language impairment [44] and better language outcomes [45] (see [46] for a general review). Our Phase 1 results indicate that the pattern of response shown by children with ASD who exhibit more severe social symptoms does not resemble the focal ERP pattern of significant difference between known and unknown words limited to the left temporal/parietal region shown by TD children in this study and in previous work [18]–[23]. Nor does it resemble the broad, bilateral response shown in very young TD children [18], [19], which is also seen in older TD children with low productive vocabularies [23].

These results confirm an association between the pattern of ERP responses to word stimuli and social function in children with ASD. The group and subgroup effects are similar to those of our earlier study using an ERP syllable discrimination paradigm in older (3–4 year old) children with ASD [10]. The results support the theoretical hypothesis that linguistic development, both in TD children and in children with ASD, is closely linked to social development (see [15], [17]). The link between language learning and social processing will be considered in the General Discussion.

## Phase 2: ERPs to Known Words and Functional Outcomes

Phase 1 of the current study revealed that the pattern of ERP response to known and unknown words in affected children exhibiting less severe social symptoms resembled that of TD controls—a focal response with enhanced negativity to known vs. unknown words that is limited to a single left parietal electrode site, P3. In addition, the significant group level differences between affected children exhibiting less severe social symptoms and TD controls on one hand, and affected children exhibiting more severe social symptoms on the other, was shown in Phase 1 to be due to the processing of known words. Phase 2 of the current study builds on these results, investigating the predictive power of the ERP response to known words at the P3 electrode site (Time 1 ERP) in the full group of children with ASD on later linguistic, cognitive, and adaptive function. We hypothesized that this defining characteristic of the more typical ERP response to words observed in children with ASD who exhibit less severe social symptoms (i.e., a strong negative response to known words at the P3 electrode site) would, in turn, have implications for functional outcome in all children with ASD.

### Results

**Figure 2:**
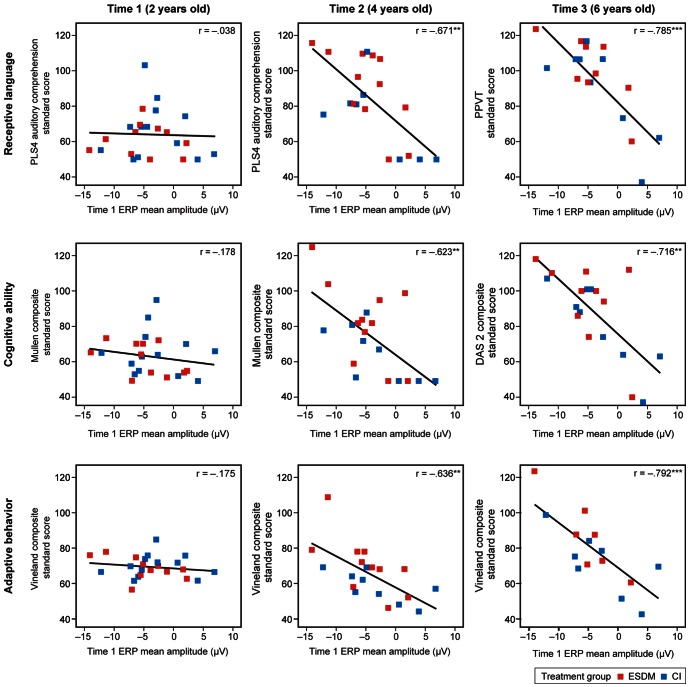
Time 1 ERP predicts later receptive language, cognitive ability, and adaptive behavior. Time 1 ERP predicts functional outcome at Time 2 (center) and Time 3 (right) in children with ASD enrolled in the ESDM (red) and CI (blue) intensive intervention groups. Time 1 ERP mean amplitude for known words (Time 1 ERP) appears on the x-axis, which is constant across all scatterplots. The specific behavioral measures of receptive language, cognitive ability, and adaptive behavior are expressed as norm referenced standard scores and appear on the y-axis. The scale for standard scores on the y-axis is constant across all scatterplots. Two-year-old children with ASD who produce a more negative Time 1 ERP amplitude have higher receptive language, cognitive ability, and adaptive behavior standard scores at the ages of 4 (Time 2, center) and 6 years (Time 3, right). Not all participants completed all behavioral measures at Time 3.

The results of Phase 2 strongly support the hypothesis that brain responses to words in children with ASD at enrollment (Time 1) predict outcome measures 2 and 4 years later when the children are 4 and 6 years of age. Standardized behavioral measures of receptive language, cognitive ability, and adaptive function were collected at enrollment (Time 1), 2 years later at the end of the experimental intervention (Time 2), and 4 years later in a follow up study (Time 3). The scatterplots in Figure 2 show the relationships between Time 1 ERP (shown on the x-axis) and measures of receptive language, cognitive ability, and adaptive function at Time 1, Time 2, and Time 3. The measures of receptive language, cognitive ability, and adaptive behavior are expressed as norm-referenced standard scores and are plotted on the y-axis using the same scale across scatterplots. Constructing the x- and y-axes in this manner allows comparison of each child (uniquely identified by Time 1 ERP mean amplitude on the x-axis) to other children in his or her age group, and allows comparison of each child's standard scores on the same scale across time and standardized tests. In these plots, the two intervention groups are differentiated by a color code: ESDM (red) and CI (blue).

Time 1 ERP was not related to Time 1 receptive language (*r* = −.038, *p*>.10), cognitive ability (*r* = −.178, *p*>.10), or adaptive function (*r* = −.175, *p*>.10) (Figure 2, left column). However, at Time 2, the Time 1 brain measure is highly correlated with standard scores for all three domains: tests of receptive language (*r* = −.671, *p*<.01), cognitive ability (*r* = −.623, *p*<.01), and adaptive function (*r* = −.636, *p*<.01) (Figure 2, middle column). At Time 3, the Time 1 brain measure is more strongly correlated with standard scores in all domains when compared to Time 2: tests of receptive language (*r* = −.785, *p*<.001), cognitive ability (*r* = −.716, *p*<.01), and adaptive function (*r* = −.792, *p*<.001) (Figure 2, right column). As shown at both Time 2 and Time 3, children with ASD who exhibited the defining characteristic of the more typical Time 1 ERP response (i.e., a strong negative response to known words at the P3 electrode site) had increasingly higher standard scores on measures of receptive language, cognitive ability, and adaptive behavior 2 and 4 years later. Examination of the scatterplots in Figure 2 indicates that the predictive relationship between the Time 1 ERP response to known words holds regardless of the assigned clinical intervention group.

Cognitive ability is a frequently reported predictor of functional outcome in children with ASD [4]–[9]. Is it possible that the link between Time 1 ERP response to words and later measures of functional outcome goes no further than expected based on the demonstrated predictive power of early cognitive ability? To examine this possibility, we first entered Time 1 Mullen Composite (a measure of cognitive ability), and then added Time 1 ERP response as a second step in linear multiple regression analyses of later receptive language, cognitive ability, and adaptive function. The results, shown in Table 5, indicate that Time 1 ERP to known words and Time 1 Mullen Composite are both significant predictors of Time 2 measures of receptive language, cognitive ability, and adaptive function, and have similar impact on the model with Time 1 ERP accounting for significant additional variance. However, the predictive power and the unique variance accounted for by the Time 1 ERP to known words is larger at Time 3, and Time 1 ERP is the sole significant predictor for standardized developmental measures of receptive language, cognitive ability, and adaptive function at Time 3 when children are 6 years of age (Table 5).

**Table 5:** Summary of linear regression analyses.

		TIME 2: 4 YEARS OLD					TIME 3: 6 YEARS OLD				
		*PLS4 Auditory Comprehension (N = 20)*					*Peabody Picture Vocabulary Test (N = 18)*				
*RECEPTIVE LANGUAGE*		B	SE B	β	R^2^	ΔR^2^	B	SE B	β	R^2^	ΔR^2^
Model 1	Time 1 Mullen Composite	1.596	.521	**.585** **	.343		1.317	.630	.463	.214	
Model 2	Time 1 Mullen Composite	1.066	.460	**.391** *			.761	.431	.268		
	Time 1 ERP	−2.253	.723	**−.526** **	.582	.239	−3.054	.651	**−.711** ***	.682	.467

		*Mullen Composite (N = 20)*					*DAS2 Composite (N = 19)*				
*COGNITIVE ABILITY*		B	SE B	β	R^2^	ΔR^2^	B	SE B	β	R^2^	ΔR^2^
Model 1	Time 1 Mullen Composite	1.497	.497	**.579** **	.336		1.201	.625	.422	.178	
Model 2	Time 1 Mullen Composite	1.045	.463	**.404** *			.554	.512	.195		
	Time 1 ERP	−1.921	.727	**−.473** *	.529	.193	−2.798	.777	**−.648** **	.546	.368

		*Vineland Composite (N = 20)*					*Vineland Composite (N = 15)*				
*ADAPTIVE FUNCTION*		B	SE B	β	R^2^	ΔR^2^	B	SE B	β	R^2^	ΔR^2^
Model 1	Time 1 Mullen Composite	1.227	.299	**.695** **	.484		.848	.554	.390	.152	
Model 2	Time 1 Mullen Composite	.940	.272	**.533** **			.512	.363	.236		
	Time 1 ERP	−1.216	.428	**−.439** *	.650	.166	−2.379	.535	**−.743** **	.680	.528

* p<.05,

** p<.01,

*** p<.001.

### Discussion

Phase 1 of the current study showed that children with ASD exhibiting less severe social symptoms demonstrate a more typical pattern of ERP response to known and unknown words; that is, they show the signature focal negativity for known words at a single left hemisphere parietal electrode site, P3. The goal of Phase 2 of the study was to investigate the relationship between this brain response to known words at the P3 site and functional outcomes in children with ASD. Thus, Phase 2 was a prospective longitudinal study that examined the power of the Time 1 brain measure in predicting behavioral outcomes for linguistic, cognitive, and adaptive function years later, when the children with ASD were 4 years of age (Time 2) and 6 years of age (Time 3).

Our Phase 2 results supported the hypothesis that a brain measure of word processing at age 2 in children with ASD predicts future linguistic, cognitive, and adaptive behavior. The Time 1 ERP measure is a strong and significant predictor of future behavior over a broad range of domains, with significant correlations between our Time 1 ERP measure and future behavioral scores on all standardized tests of linguistic, cognitive, and adaptive behavior at Time 2 when the children with ASD were 4 years of age, and at Time 3 when the children with ASD were 6 years of age. As the scatterplots of Figure 2 reveal, all children with ASD exhibiting a strong negative response to known words at the P3 electrode site are much improved by Time 3, with scores in the average or near-average range. In contrast, affected children exhibiting a less typical response to known words at Time 1 show less improvement over time. Comparing the significant correlations shown at Time 2 with those shown at Time 3 indicates that our Time 1 ERP predictor becomes stronger over time. In addition, the predictive relationships seen at Time 2 and Time 3 hold regardless of treatment group assignment of the children with ASD.

Comparing a known predictor of later function in children with ASD (cognitive ability) [4]–[9] and our ERP brain response to known words was also a goal of Phase 2. Both Time 1 cognitive ability and Time 1 ERP are significant predictors of function at Time 2, consistent with previous literature. However, our results show that cognitive ability is no longer a significant predictor of behavioral outcomes at Time 3. In contrast, the predictive power of Time 1 ERP persists over time and generalizes across the different standardized tests of language and cognition administered at Time 3.

The lack of concurrent correlations between the Time 1 ERP measure and Time 1 performance on standardized measures seen in Experiment 2 is consistent with observations reported in longitudinal studies of language development. Previous work in TD populations demonstrating that measures of early language processing predict future language outcomes [24]–[33] also shows that predictive relationships can occur in the absence of concurrent relationships. For example, Fernald et al. [30] measured lexical/grammatical competence and word processing efficiency in TD children at 12, 15, 18, 21 and 25 months, and found significant prospective, predictive relationships between lexical/grammatical competence and word processing speed that were not observed concurrently. The results of the current study's Phase 2 are consistent with this finding: Time 1 ERP is not related to the Time 1 behavioral scores, while strong and significant correlations emerge at Time 2 and become stronger at Time 3. Authors of the previous studies [24]–[33] interpret their results as evidence that many early abilities interact during the developmental process providing the scaffold for a synergistic learning process that unfolds over time, and therefore concurrent correlations may not be found, even when prospective correlations are very strong, as shown in the results obtained in Phase 2.

We believe that our ERP measure of word learning at Time 1 reflects the coupling of specific computational skills and social skills, and may be time sensitive. Furthermore, the presence of this neural indicator at Time 1 may signal the opportunity for advancement toward higher linguistic and cognitive functions (see [15], [16] and General Discussion), consistent with the interpretations of previous research.

### Limitations of the Study

Aspects of the current study suggest caution in the interpretation of our results. First, participants were restricted to children from monolingual English-speaking families, as was most previous work with this ERP word paradigm. Second, meta-analysis of attrition rates in EEG studies on young children show an average attrition rate of 47.3%, comparable to the attrition rate shown in the current study, which was 40% among children with ASD. High attrition rates reduce the utility of potentially informative predictive measures. We also note, however, that affected children with and without usable ERP data did not differ significantly in terms of age, gender, diagnostic measures, standardized measures of receptive language, cognitive ability, or adaptive behavior at Time 1, or in assignment to intervention groups. Consequently, the prediction of outcomes in children diagnosed with ASD at the age of two years should theoretically be possible in the broader population of children with ASD if obstacles to obtaining usable ERP can be overcome. Third, small sample sizes such as ours (N = 24) require cautious interpretation of the results and may not extend to larger samples of subjects. Fourth and most important, it is critical to note that all participants in the current study received some form of intensive treatment—we did not test children with ASD who received no treatment. Our findings apply to children with ASD who received some form of intensive clinical treatment.

## General Discussion

The results of the present study provided two new findings. First, Phase 1 of the study revealed an important link between brain measures of word processing and social function in very young children with ASD. Children with ASD who exhibit less severe social symptoms have a pattern of ERP response to known and unknown words that resembles that of TD controls, whereas affected children with more severe social symptoms show an atypical pattern. Taken together with our previous result showing subgroup effects in children with ASD for phonetic stimuli, the current results on words reinforce the theoretical view that the early acquisition of language is tightly coupled to social function in TD children [14], [17], [47], [48] and in children with ASD [49]–[51].

Second, Phase 2 of the current study revealed that the identified signature for known words in the children with ASD at age 2 years is a powerful predictor of linguistic, cognitive, and adaptive outcomes in all children with ASD many years later, when the children were 4 years of age, and when these same children were 6 years of age. This result was obtained controlling for cognitive ability of the children with ASD at enrollment, and independent of the specific of intensive clinical treatment during the intervening years of the randomized clinical trial [34]. These findings have implications for theories of language development as well as translational impact on research in the arena of ASD.

From a theoretical standpoint, the results reported here support arguments that the early period of linguistic development is extremely important in setting the stage for future development. In studies of TD children, the initial learning of phonemic units that are relevant for the native language predicts future language development [16], [24]–[33], [52]. A decade ago, there were no prospective longitudinal studies linking early speech perception to later language in TD children (see [31] for review). At present, many studies of TD children show that variations in early measures of language learning are not ‘noise,’ but instead meaningful indices that predict the speed of future language growth in individual children. The present study extends this prospective longitudinal design to children with autism, revealing for the first time that a brain measure of word processing at the age of 2 in children with ASD predicts future growth in language, cognition, and adaptive behavior up to 4 years later, exceeding the predictive value of cognitive ability, which has been associated with outcomes in children with ASD. These findings encourage the use of brain measures earlier in the development of children at risk for ASD, and offer the promise that highly sensitive brain measures of language processing may provide information on developmental trajectories in children with ASD. We are a long way from identifying a neural indicator that would predict a future diagnosis of ASD, but the present findings and recent studies relating neural characteristics in infants to later diagnosis of autism [53], [54] suggest that neural indices could some day serve this role.

In addition, the present results buttress the theoretical argument linking social and linguistic processing [15], [17]. Work in this laboratory and others has advanced the hypothesis that computational mechanisms, which involve extracting information about the statistical relationships in language from language input, underlie language learning. For example, laboratory experiments in TD infants show that phonetic learning is advanced by infants' sensitivity to the distributional frequency of phonetic units in language input (see [13], [55]). Infants learn the phonetic units with the highest frequency of occurrence. Similarly, early word learning is advanced by infants' sensitivity to the probability that one syllable will follow another in language input: two syllables that follow each other frequently (like the ‘ba’ and ‘by’ of ‘baby’) are treated as a likely word, and syllables that do not follow each other frequently are treated as nonwords [56]. The discovery of these computational skills in infants constituted an important advance in understanding the mechanisms by which language learning progresses. But equally important to theory are more recent experiments that suggest a constraint on infants' computational learning: To use these computational mechanisms for natural language learning, infants appear to require a social context; that is, they learn only when interacting with a live human being [14]. These lines of research led to the hypothesis that the social brain ‘gates’ the computational mechanisms of early language learning [14], [15]. The implications of these findings from TD children for children with ASD are clear. If the social brain ‘gates’ learning, then children with ASD will be at a strong disadvantage in the acquisition of early language. Applying this theoretical formulation to children with autism suggests that classifying children with ASD on measures of social function should produce very different patterns of brain response to linguistic stimuli in the two subgroups of children with ASD. The subgroup of children with ASD who exhibit less severe social impairment should produce patterns of brain response to linguistic stimuli that more closely resemble the patterns exhibited by TD children, whereas the subgroup of children with ASD who exhibit more severe social impairment should produce a more atypical pattern. Our current study's Phase 1, employing word stimuli, and our previous result employing phonetic stimuli [10], provide support for this hypothesis.

One premise underlying the deep theoretical links between social and linguistic processing is ‘sensitive periods’ in language learning— which asserts that initial learning of native-language phonemes, words, and grammatical structure is time sensitive [16]. Broad proof for this claim is to be found in the many studies on second language acquisition in which learning before the age of 7 years is far superior to learning that occurs later. Research on phonetic learning strongly suggests a sensitive period (see [16], [57]), and similar data exist to suggest a sensitive period for grammatical learning [58].

The evidence that word learning is time sensitive is less clear. The data in TD children suggests that, at approximately 2 years of age, reorganization occurs in the brain for word processing. It is at this time that the acceleration in expressive vocabulary growth has been documented in many of the world's languages [59]–[61]. During this time period, differential ERP responses to known and unknown words become more focal, with significant differences in the temporal/parietal region of the left hemisphere in typical populations, as well as in late talkers, and, as shown here, in the subgroup of children with ASD who exhibit less severe social symptoms. Mills and colleagues interpret this developmental change in ERP response to words in TD children as resulting from a dynamic organization of language-relevant brain activity in which multiple, continuous developmental processes (i.e., experience with individual words, experience with general word learning, increases in working memory) allow more efficient word processing accompanied by more focal differential responses to known and unknown words in the left hemisphere (see [23] for review). The social gating hypothesis [15], [24] predicts that the interaction of social factors and language input operate in concert to advance the development of word processing in TD populations as described above. Our results suggest that in children with ASD, the synergistic effects of social and linguistic function are disrupted. Individual differences in severity of social symptoms have demonstrable effects on ERP response to words such that the subgroup of affected children with less severe social symptoms exhibit the focal response to words in the left hemisphere. We suggest that the focal left hemisphere ERP response to words observed in TD children, as well as in a subgroup of children with less severe social symptoms of ASD, is an early manifestation of more efficient word processing, and therefore serves as a powerful predictor of later function in *all* children with ASD.

Our work shows that phonetic learning in TD children between 6 and 12 months of age also predicts the speed with which they acquire language; better native language discrimination predicts faster growth of language to the age of 30 months [24], [31]. Importantly, our results demonstrate that the significant predictor of future language growth in TD children is phonetic *learning*, not simply skill at phonetic discrimination, because the ability to discriminate nonnative phonetic contrasts in the same infants, measured at the same time as native discrimination, also predicts future language, but in the opposite direction. The better infants are at nonnative discrimination the slower future language develops [24], [31]. In other words, the growth of language in young children depends on their ability to attend to the linguistically relevant phonetic distinctions in social contexts, rather than attending to all phonetic distinctions.

Our present findings in 2-year-old children with ASD regarding word processing may suggest a similar social learning process. The Time 1 ERP measure of known words is highly predictive of future abilities in affected children. The predictive value of the Time 1 ERP measure in children with ASD increases with time and goes beyond the predictive power of a traditional cognitive measure. The defining characteristic of a focal response to words in the left hemisphere may be indicative of the neural structures necessary to support efficient learning of more complex linguistic structures, advanced cognitive skills, and the development of appropriate adaptive behaviors in children with ASD. It is possible that this neural indicator of word learning reflects the same type of learning-based neural reorganization we see in phonetic development [13], [16]. If so, and many more experiments will be required to support this conclusion, it will be important to ascertain whether a reorganization for word learning is time sensitive. To the extent that aspects of language learning are time sensitive, early diagnosis of ASD is vitally important, allowing treatment interventions as early in development as possible.

The findings of the present study also have translational impact on research in the arena of ASD. First, the finding that a neural measure of linguistic learning at 2 years of age can successfully predict broad behavioral outcomes 4 years later in children with ASD encourages further explorations into neural measures that can be applied even earlier in development. In the current study, children with ASD were 2 years of age. The ERP word paradigm used here has been applied to children as young as 13 months of age [19] and we are now pursuing the goal of applying this paradigm in current research in younger children at risk for ASD using a brain measure that can identify sources of neural activation through MEG technology [16].

A caveat regarding the present results stems from the finding that our Time 1 measure predicted outcomes broadly in children with ASD regardless of the form of intensive treatment they received. It is important to note that all participants in the current study received some form of intensive treatment—we did not test a group of children with ASD who received no treatment. Our findings apply to children with ASD who are tested at the age of 2 years, and receive some form of intensive clinical treatment. Under these conditions, we show that, regardless of the form of intensive treatment the children received, our ERP measure predicts outcomes on standardized measures of linguistic, cognitive, and adaptive behavior, as long as 4 years after the initial assessment.

In conclusion, we show that ERP measures based on word processing in children with ASD who have been grouped on a variable that assesses social function provides an excellent neural indicator of future function across the domains of language, cognition, and adaptive function up to 4 years after the initial assessment. The working hypothesis is that our neural indicator reveals reorganization in the brain's processing of words and that this reorganization reflects the ability of the brain to learn from social experience. The ERP measure used in the current study (a) exceeds the predictive value of a cognitive measure assessed at the same initial point in time, (b) becomes increasingly predictive over time, and (c) holds regardless of the type of intensive treatment received by the children with ASD during the intervening 4 years. Neural indicators of language function have the potential to be assessed earlier in development. Identification of an early language-based prognostic brain measure for children with ASD holds promise for novel early intervention methods that are tailored to individual children, and may enhance outcomes for all children with ASD.
